# Cutoff Values of Hemodynamic Parameters in Pediatric Refractory Septic Shock

**DOI:** 10.3390/children9030303

**Published:** 2022-02-22

**Authors:** En-Pei Lee, Jainn-Jim Lin, Shao-Hsuan Hsia, Oi-Wa Chan, Sheng-Ling Jan, Han-Ping Wu

**Affiliations:** 1 Division of Pediatric Critical Care Medicine, Department of Pediatrics, Chang Gung Memorial Hospital at Linko, Kweishan, Taoyuan 333, Taiwan; pilichrislnp@gmail.com (E.-P.L.); lin0227@cgmh.org.tw (J.-J.L.); tw1picu002@gmail.com (S.-H.H.); oiwamail@gmail.com (O.-W.C.); 2 College of Medicine, Chang Gung University, Taoyuan 333, Taiwan; 3 Department of Pediatrics, Children’s Medical Center, Taichung Veterans General Hospital, Taichung City 407, Taiwan; 4 School of Medicine, National Yang-Ming Chiao-Tung University, Taipei City 112, Taiwan; 5 School of Medicine, Kaohsiung Medical University, Kaohsiung 80708, Taiwan; 6 Department of Pediatric Emergency Medicine, Children’s Hospital, China Medical University, Taichung 406040, Taiwan; 7 Department of Medical Research, Children’s Hospital, China Medical University, Taichung 406040, Taiwan; 8 Department of Medicine, School of Medicine, China Medical University, Taichung 406040, Taiwan

**Keywords:** cutoff values, hemodynamic parameters, pediatric septic shock, outcomes, intensive care unit

## Abstract

Background: Refractory septic shock can cause severe morbidities and mortalities in children. Resuscitation based on hemodynamics is important in children with critical illness. Thus, this study aimed to identify the hemodynamics of refractory septic shock associated with poor prognosis at an early stage to allow for timely interventions. Methods: We evaluated children with refractory septic shock admitted to a pediatric intensive care unit (PICU) and monitored their hemodynamics using a pulse index continuous cardiac output (PiCCO) system. The serial cardiac index (CI), systemic vascular resistance index (SVRI), and vasoactive–inotropic score (VIS) were recorded during the first 72 h after PICU admission. Results: Thirty-three children with refractory septic shock were enrolled. The SVRI and VIS were both associated with fatality from septic shock. The non-survivors had lower serial SVRI and higher VIS (both *p* < 0.05). Based on the area under the ROC curve, the SVRI was the predictor during the early resuscitative stage (first 36 h) in pediatric refractory septic shock. Conclusions: Both SVRI and VIS are predictors of mortality in children with refractory septic shock, and the SVRI is the powerful predictor of mortality in the early resuscitative stage. A low serial SVRI may allow for the early awareness of disease severity and strategies for adjusting vasoactive–inotropic agents to increase the SVRI.

## 1. Introduction

Circulatory shock refers to inappropriate perfusion that results in damage to tissues and causes mortality in children, which accounts for one-third of cases in intensive care units. According to the International Consensus Conference on Pediatric Sepsis, sepsis is defined as the systemic inflammatory response to a suspected or proven infection [1,2], and it can be graded on the basis of the severity and response to therapy, such as severe sepsis, septic shock, and refractory septic shock (RSS) [2]. The severity of sepsis from mild to severe was described previously, and the respective definitions are described as follows: severe sepsis (cardiovascular dysfunction, acute respiratory distress syndrome, or dysfunction in ≥2 other organ systems), septic shock (sepsis with cardiovascular dysfunction which need vasoactive medication despite isotonic fluid resuscitation), and RSS (circulatory failure caused by septic cardiomyopathy despite fluid and vasoactive agents treatment) [2,3].

In children, sepsis remains the main cause of morbidity and mortality worldwide, and RSS is the most severe form of sepsis for which the rate of fatality ranges from 40% to 80% [4,5]. At present, the Surviving Sepsis Campaign 2020 published the clinical standard for hemodynamic maintenance in children with sepsis [6]. In pediatric RSS, the important hemodynamic parameters are the cardiac index (CI) and systemic vascular resistance index (SVRI). The therapeutic goal of CI 3.3–6.0 L/min/m^2^ may help achieve better outcomes [6,7,8]. Some reports have indicated that abnormal SVRI is associated with poor outcomes [9,10]. Furthermore, the vasoactive–inotropic score (VIS) is an equitable way of determining whether an individual requires vasoactive–inotropic drugs for cardiovascular support, which is also associated with prognosis [3,10]. Therefore, detecting abnormal hemodynamics in case of RSS at an early stage is important to allow for timely interventions with new vasoactive–inotropic agents to maintain threshold hemodynamics and improve clinical outcomes [11,12].

Few studies have investigated the systemic hemodynamics and VIS associated with 28-day mortality in children with RSS, and to our knowledge, no study has compared the performance of major hemodynamics, such as CI, SVRI, and VIS, to predict mortality in children with sepsis.

The pulse index continuous cardiac output (PiCCO) system incorporates transpulmonary thermodilution (TPTD), and it has shown effectiveness in monitoring the hemodynamics in children with critical illness. In this study, we aimed to analyze correlations between 28-day mortality and the most important hemodynamic parameters (i.e., serial CI and SVRI) and serial VIS in children with RSS.

## 2. Materials and Methods

### 2.1. Patient Population

Chart reviews of pediatric patients aged <18 years who had shock in a PICU were conducted from 2003 to 2017. A total of 29 beds were available in our PICU, and we treated patients aged from 1 month to 18 years. The study criteria, based on consensus definitions, were applied uniformly in our PICU, resulting in an internal standardized evaluation of the study [2].

Chang Gung Memorial Hospital’s Institutional Review Board and Ethics Committee approved this study.

### 2.2. Study Design

The inclusion criterion was as follows: children with RSS who were admitted to the PICU. Fluid-RSS was constant shock regardless of the 60 mL/kg of fluid resuscitation according to the guidelines of the Surviving Sepsis Campaign 2012 [3,13,14]. Catecholamine-resistant shock was constant shock after fluid resuscitation and receiving one vasoactive medication [2,15]. Persistent catecholamine-resistant shock was constant shock despite receiving two kinds of vasoactive medications. Children with persistent catecholamine-resistant shock were enrolled, and the hemodynamics was monitored via a PiCCO system (Pulsion Medical Systems, Munich, Germany). We gathered data pertaining to our patients: age; sex; baseline cardiac characteristics, such as initial inotropic equivalent, heart rate (beats/min), and mean arterial pressure (MAP; mmHg); PiCCO system parameters; length of stay; and mortality.

Patients who met the criteria of persistent catecholamine-resistant shock were included, and they underwent PiCCO monitoring. Initial parameters included 72 h serial CI, SVRI, and VIS data, which were detected within 4 h of enrollment after PiCCO setup. Hemodynamics and VIS were recorded hourly after the PiCCO system was implemented. Hemodynamic parameters were further analyzed between the survivors and non-survivors to investigate whether serial CI, SVRI, and VIS could be early indicators for mortality in pediatric RSS. The two major clinical outcomes were defined as the 28-day mortality rate in the PICU and the length of ICU stay.

### 2.3. Therapeutic Strategy

We followed the guidelines of early goal-directed therapy (NEJM 2001) for children with septic shock. Resuscitation aimed to preserve the central venous pressure (CVP) of 8–12 mm Hg, adequate age-specific blood pressure (BP), cardiac index (CI) of 3.5–5.5 L/min/m^2^, and central venous saturation (ScvO2) ≥ 70%. Fluid resuscitation (10–20 mL/kg) is administered when the CVP level decreases; dopamine is administered when the systolic BP is not optimal (less than 5th percentile for age), and inotropic agents are administered when the CI level declines, ScvO2 falls below 70%, or serum lactate increases (≥2 mmol/L) [6,16].

### 2.4. PiCCO Parameter Measurement

Hemodynamics were continuously analyzed and output to a computer using PiCCO-VoLEF Data Acquisition software (version 6.0; Pulsion Medical Systems, Bayern, Germany).

### 2.5. Definition

The VIS was defined as dopamine dose (mcg/kg/min) + dobutamine dose (mcg/kg/min) + 100 × epinephrine dose (mcg/kg/min) + 100 × norepinephrine dose (mcg/kg/min) 10 × milrinone dose (mcg/kg/min) + 10,000 × vasopressin dose (units/kg/min) [17].

### 2.6. Statistical Analysis

Multivariate logistic regression analysis, the chi-square test, Fisher’s exact test, Student’s *t*-test, and the Mann–Whitney U test were all conducted. Descriptive analysis used means ± standard deviations to show the value. The chi-square or Fisher’s exact tests were conducted for comparison of dichotomous variables between the two groups. As a final step, the optimal cutoff values of hemodynamic parameters to predict mortality were determined using the receiver operating characteristic (ROC) curve. LR+ and LR− were calculated to obtain the best cutoff values. Likewise, DeLong’s test was conducted to determine whether a significant difference in AUCs of the hemodynamic parameters exists. Significance was set at *p* < 0.05. Statistics were conducted utilizing SPSS (version 22.0; SPSS Inc., Chicago, IL, U.S.A.).

## 3. Results

### 3.1. Demographic Characteristics of Pediatric Septic Shock

**Figure 1:**
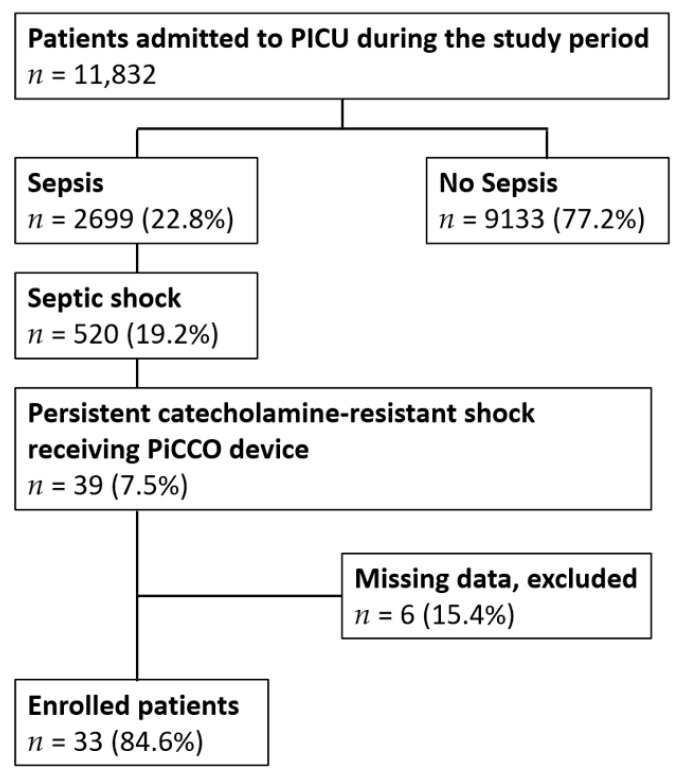
Algorithm of enrolled patients.

Over the course of the 15-year study period, 11,832 patients were admitted to our PICU, of whom 2699 (22.8%) suffered from sepsis. Septic shock was observed in 520 (19.2%) of the 2699 patients with sepsis. The PiCCO device was inserted for invasive hemodynamic monitoring in 39 children with persistent catecholamine-resistant shock. As six patients had missing data, we finally enrolled 33 patients for further analysis (Figure 1). Among the enrolled children, 72.8% were microbiologically confirmed as having an infection. The bloodstream was the most frequently observed site of infection, with Gram-negative bacteria as the predominant pathogen. The patient survival rate was 45%, and the mortality rate was 55%. Both survival and mortality groups commonly used dopamine and epinephrine as initial vasoactive–inotropic agents. After the PiCCO arrangement, the non-survivors exhibited a significantly higher VIS but a significantly lower MAP, compared with the survivors (*p* < 0.05).

### 3.2. PiCCO Parameters after Setting Up TPTD

CO and cardiac contractility parameters measured by PiCCO showed no significant difference between survivors and non-survivors (Table 1). A higher SVV (a preload parameter) and lower SVRI (an afterload parameter) were observed in the non-survivors compared with the survivors (both *p* < 0.05).

### 3.3. Factors Associated with 28-Day Mortality

**Figure 2:**
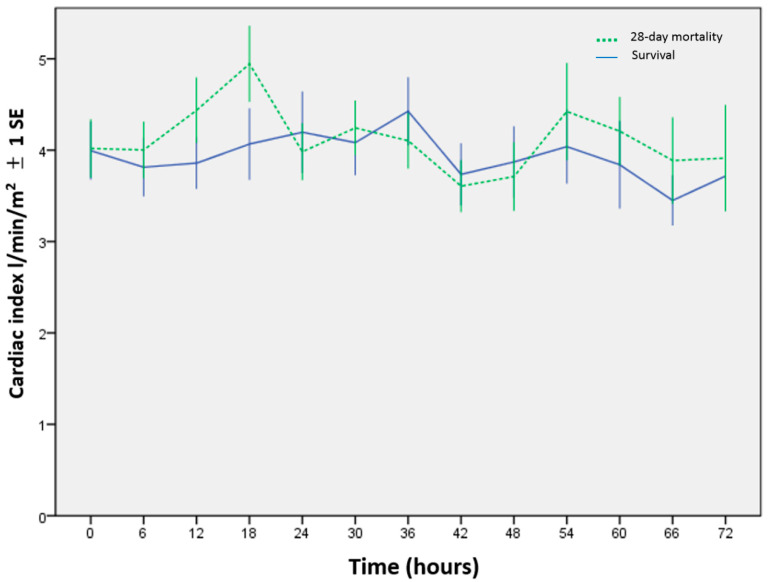
Serial cardiac index (CI) and its variance (mean ± 1 SE bar) after comparing survivors with non-survivors, measured at 6 h intervals.

**Figure 3:**
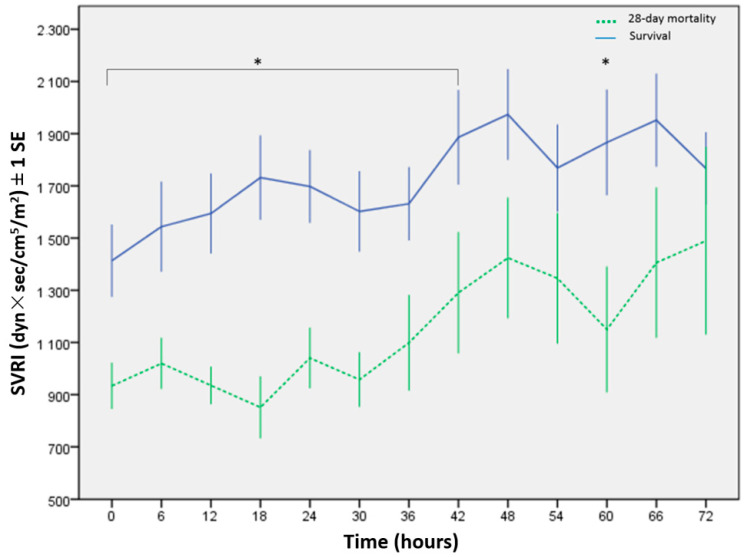
Serial systemic vascular resistance index (SVRI), measured at 6 h intervals. * Statistical significance was set at *p* < 0.05.

**Figure 4:**
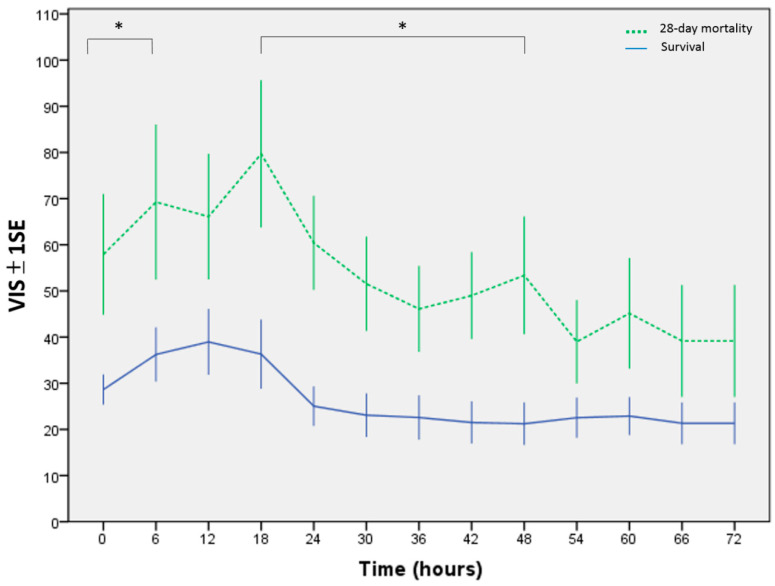
Serial vasoactive–inotropic score (VIS), measured at 6 h intervals. * Statistical significance was set at *p* < 0.05.

**Figure 5:**
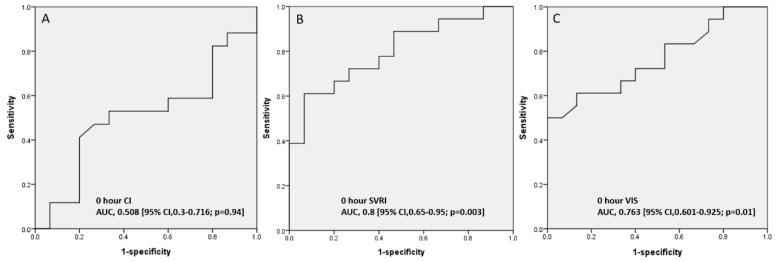
Predicting 28-day mortality at 0 h based on the AUCs of CI, SVRI, and VIS. (**A**) CI, (**B**) SVRI, and (**C**) VIS.

**Figure 6:**
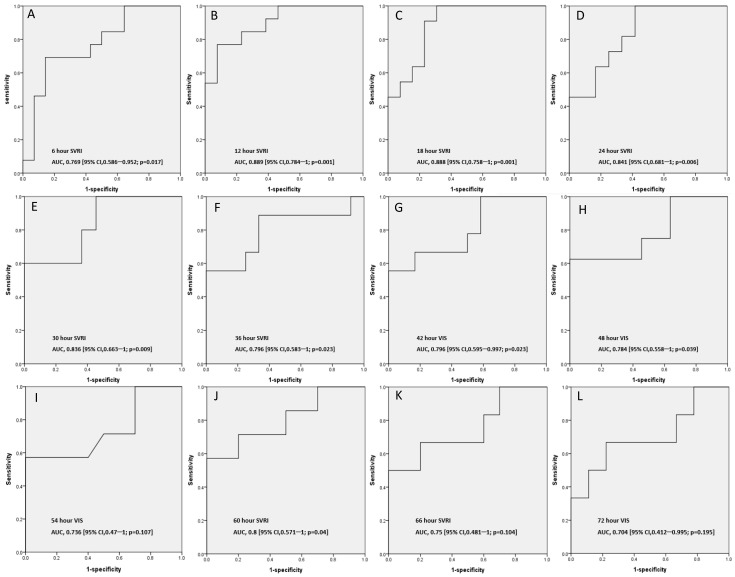
Predicting 28-day mortality at 6 h intervals using the best AUC. (**A**–**L**) 6–72 h.

After the PiCCO device were implemented, the CI was measured every 6 h (Figure 2). Only 33 cases were identified in this study; thus, three important parameters (CI, SVRI, and VIS) were put in the multivariate logistic regression analysis. The results reported that the SVRI and VIS were independent predictors of 28-day mortality during the first 72 h after admission to the PICU (Table 2). Table 3 summarizes the AUC and cutoff values of CI, SVRI, and VIS for the survival and mortality groups every 6 h. Within the first 36 h after patients were enrolled, the average AUC of the SVRI was the highest (0.83) among all predictors, and within 42–54 h, the VIS showed the highest average AUC (0.77); however, within 60–66 h, the SVRI showed the highest average AUC (0.78). The serial SVRI measured every 6 h was lower in the non-survivors, which was significantly lower within 0–42 h and at the 60th hour (Figure 3). The serial VIS was higher in the mortality group, particularly within 0–48 h, except at the 12th hour (Figure 4). Figure 5 and Figure 6 depict the ROC analysis for CI, SVRI, and VIS calculated every 6 h when attempting to predict survival, and Table 4 provides the best CI cutoff values for each predictor. DeLong’s test showed that the AUC of the SVRI was better than that of the CI (*p* < 0.05), but no significant difference was noted between VIS and CI. Furthermore, the two cutoff values of 100% specificity and 100% sensitivity for the SVRI in predicting clinical outcomes at 6 h interval are shown in Table 5.

## 4. Discussion

Septic shock has a high in-hospital mortality rate, particularly for children who experience RSS. Hemodynamic monitoring is essential for the evaluation and therapeutic management of patients with critical illness, especially for children. In this 15-year retrospective clinical study, the SVRI and VIS were all independent predictors of 28-day mortality in pediatric RSS. A lower SVRI and a higher VIS were noted at similar time points in the non-survivors. Furthermore, we identified that SVRI has the most predictive power compared to CI and VIS during the early resuscitative stage of refractory septic shock.

During the early stage of septic shock, clinical presentations may include warm skin, tachycardia, and widened pulse pressure, and warm shock may indicate the hemodynamics of increased CI and decreased SVRI [13]. However, as sepsis progresses, cold shock may develop with decreased CI and increased SVRI. Our serial data showed a clinical course of higher CI along with lower SVRI, and a gradual decrease or normalization in CI accompanied with an increasing SVRI. Interestingly, under vasoactive–inotropic agent support, the average serial CI in the mortality group in our cohort reached a normal range of 3.3–6.0 L/min/m^2^, which is different to the American College of Critical Care Medicine guidelines, which state that a normal CI indicates a good prognosis [14].

In patients with sepsis, a low SVRI may indicate decreased vasomotor tone caused by endothelial injury, followed by decompensation, leading to an increase in CI levels [18]. Injured endothelial cells indicate dysfunction of the arginine–vasopressin system, which may increase the secretion of tumor necrosis factor, lipopolysaccharides, interleukin-1, circulating endothelin, and nitric oxide, and decrease systemic vascular resistance, causing vascular hyporesponsiveness to vasoactive agents, thereby resulting in refractory hypotension [19]. Previous reports have shown that lower SVRI is associated with mortality in adults with septic shock [20,21]; however, the clinical utilization of SVRI has not been well surveyed in pediatric septic shock. Our previous study with the same group reported that SVRI was a predictor for in-hospital mortality in pediatric septic shock [22]. The current study was an expansion of the previous one, which demonstrated the importance of SVRI in the different time points of pediatric septic shock. Our study demonstrated that serial SVRI was significantly lower in the non-survivors. Therefore, early higher levels of SVRI may indicate better outcomes. In the mortality group, although the SVRI increased gradually, the prolonged period of a low SVRI indicated a longer duration of tissue hypoperfusion, representing more organ damage [20]. Moreover, lower serial SVRI may allow the early recognition of disease severity and insufficient treatment and prompt the immediate titration of vasoactive–inotropic agents to raise the SVRI.

Administration of vasoactive–inotropic agents to resuscitate fluid-unresponsive septic shock remains the standard treatment [15]. The VIS may allow for the evaluation of the degree of cardiovascular support, and a high VIS may indicate serious myocardial dysfunction and vasoplegia and serve as a predictor of poor clinical outcomes in children after cardiothoracic surgery and RSS [3,23]. Most patients in both groups used epinephrine or dopamine as the first vasoactive–inotropic agent; however, the VIS quickly increased in the mortality group, and serial VIS was higher. In addition, the clinical benefits of the VIS may include the ease of calculation without the need to analyze prior medical records.

During the first 36 h, the average AUC of SVRI was higher than that of VIS; however, during the period from 42 to 54 h, the result was reversed. The different cutoff values of the SVRI or VIS to predict mortality every 6 h might confuse physicians when and how to use them probably because of the clinical course of vasoplegia. A significant correlation was observed between the SVRI and VIS, which indicates that vasoplegia could be a predictive factor of septic shock. Vasoplegia was defined as vascular hyporesponsiveness to vasopressors [24]. The symptoms of vasoplegia were commonly observed without thorough hemodynamic data, and the parameters available to clinicians were not sufficient to identify the severity and implications of the condition. [25]. In the present study, we graded the severity of vasoplegia based on the SVRI and VIS. Vasoplegia is a severe condition characterized by persistent vasodilatation despite the use of vasoactive–inotropic agents at high doses. Therefore, the hemodynamics of severe vasoplegia may have low SVRI but high VIS; conversely, mild vasoplegia may have high SVRI but low VIS. Our data demonstrated that low SVRI may initially predict mortality (during the first 36 h). Although the SVRI increased gradually (after 36 h), children with increasing SVRI under high doses of vasopressors (higher VIS) had mortality (from 42 to 54 h). Given the opposite relationship between SVRI and VIS, the intensivists may interpret that the two hemodynamic parameters can predict the progression of vasoplegia in septic shock.

In clinical terms, the cutoff SVRI is divided into three zones: the first zone is for predicting the highest likelihood of mortality (specificity, 100%), the second zone is for predicting survival (sensitivity, 100%), and the third zone is indeterminate. The SVRI in predicting mortality at 0 h was 533 (specificity, 100%), and the SVRI in predicting survival at 0 h was 1531 (sensitivity, 100%). Most children with SVRI < 500 after critical care may indicate a high probability of mortality, whereas most children with SVRI > 1500 may indicate a high probability of survival. The recommended range of SVRI should be more than 500, and α-adrenergic agonists, such as epinephrine or norepinephrine, should be titrated immediately to prevent SVRI < 500. The SVRI in the survivors tended to increase after intensive care. Gradual decreases in SVRI after treatment in the indeterminate zone should be considered alarm signs that require immediate re-evaluation to identify whether or not other factors are present, such as worsening end-organ hypoperfusion and uncontrolled infectious sources.

This study has several limitations. First, this study was executed at a single center with a retrospective design and relatively small sample size. Therefore, information bias may occur. Second, incomplete data regarding macrohemodynamic parameters, such as systolic and diastolic pressures, fluid balance, need for hemodialysis, and serum lactate, were lacking. The lack of this information may prevent the external validation of the results of this study. Future studies should address the questions and verify these results prospectively. Third, the PiCCO device needs one central venous catheter and one femoral artery catheter. Placing the femoral artery catheter in younger children is difficult because the diameter of arteries is smaller than that in adults, particularly when they are in shock status. Therefore, we only included older children with the mean age of 12 years, and the youngest patient in this study was 5 years old. Future studies using noninvasive continuous CO monitoring tools, such as electrical cardiometry, are warranted to verify hemodynamics in neonates and children with septic shock. Fourth, although the Surviving Sepsis Campaign 2020 was recently published, the study period ranged from 2003 to 2017, so the definitions (refractory shock, catecholamine-resistant shock and persistent catecholamine-resistant shock) were based on Surviving Sepsis Campaign 2012, which are the same as Surviving Sepsis Campaign 2020.

## 5. Conclusion

The SVRI and VIS were predictors of mortality in children with catecholamine-RSS. In this study, the SVRI was a powerful predictor of mortality in the early resuscitative stage. Lower serial SVRI may allow for the early recognition of disease severity and strategies to titrate vasoactive–inotropic agents to increase the SVRI.

## Figures and Tables

**Table 1:** Population characteristics and initial PiCCO parameters.

Variables	Survival (n = 15)	28-Day Mortality (n = 18)	*p* Value
**Age** (years)	12.1 ± 4.6	12.3 ± 4.3	0.786
**Gender** (male), n (%)	6 (40)	10 (55.6)	0.373
**Weight** (kg)	34.5 ± 15.1	36.6 ± 15	0.703
**Underlying**, n (%)	8 (53.3)	13 (72.2)	0.446
**PRISM score**	18.3 ± 4.9	19.7 ± 3.1	0.329
**Site of infection**, n (%)			0.18
CNS	0	5 (27.8)	
Bloodstream	6 (40)	7 (38.8)	
Respiratory	4 (26.6)	3 (16.7)	
Urologic	1 (6.7)	0	
Abdominal	1 (6.7)	0	
Others	3 (20)	3 (16.7)	
**Culture positive**, n (%)	11 (73.3)	13 (72.3)	0.77
Pathogen, n (%)			0.513
Gram positive	5 (33.3)	2 (11.1)	
Gram negative	5 (33.3)	8 (44.4)	
Fungus	1 (6.8)	1 (5.6)	
Virus	0 (0)	2 (11.2)	
Unknown	4 (26.6)	5 (27.7)	
**Used vasoactive–inotropic agents**, n (%)			
Dopamine	14 (93.3)	18 (100)	0.9
Epinephrine	12 (80)	17 (94.4)	0.46
Norepinephrine	5 (33.3)	9 (50)	0.543
Dobutamine	1 (6.7)	7 (38.9)	0.08
Milrinone	10 (66.7)	10 (55.6)	0.76
Vasopressin	0	1 (5.5)	0.9
**Resuscitative IV fluid** (mL/kg)			
IV fluid prior to the study included	59.7 ± 5.3	60.1 ± 6.2	0.81
IV fluid included in the study within 72 h	17.2 ± 4.6	15.5 ± 1.2	0.164
**Cardiac characteristics**			
VIS	27.5 ± 12.3	54.7 ± 56	0.015
Heart rate (beats/min)	120.3 ± 29.9	142.4 ± 24.7	0.057
Mean arterial pressure (mm Hg)	83.5 ± 16.2	58.8 ± 16.7	<0.001
**Outcomes**			
ICU stay (days)	22.1 ± 15.7	13.8 ± 7.7	0.057
Length of stay (days)	37.3 ± 28.1	22.3 ± 16	0.093
**Initial PiCCO data**			
**Cardiac output**			
CO (L/min)	4.4 ± 1.7	4.7 ± 1.7	0.628
**Cardiac contractility**			
CI (L/min/m^2^)	3.9 ± 1.1	4.1 ± 0.9	0.602
GEF (%)	31.3 ± 10.1	27.8 ± 9.9	0.502
CFI (I/min)	9.2 ± 2.8	9.4 ± 3.4	1
**Preload parameters**			
GEDVI (mL/m^2^)	451.8 ± 154.2	457.5 ± 145.2	0.823
ITBVI (mL/m^2^)	564.2 ± 192.8	571.5 ± 181.5	0.823
SVV (%)	9.7 ± 3.3	16.1 ± 6.7	0.003
**Afterload parameter**			
SVRI (dyn×sec/cm^5^/m^2^)	1413.1 ± 537.7	933.9 ± 376.9	0.003
**Lung parameters**			
EVLWI (mL/m^2^)	10.7 ± 4.7	15.9 ± 14.9	0.551
PVPI	2.9 ± 0.9	3.9 ± 3.1	0.502

PRISM, pediatric risk of mortality; CNS, central nervous system; IV, intravascular. VIS, vasoactive-inotropic scores; ICU, intensive care unit; CO, cardiac output; CI, cardiac index; GEF, global ejection fraction; CFI, cardiac function index; GEDVI, global end-diastolic volume index; ITBVI, intrathoracic blood volume index; SVV, stroke volume variation; SVRI, systemic vascular resistance index; EVLWI, extravascular lung water index; PVPI, pulmonary vascular permeability index.

**Table 2:** Multivariable analysis for the predictors of the 28-day mortality during the first 72 h after admission to the PICU.

Parameter	β	Odds Ratio	*p* Value
**CI** (L/min/m^2^)	−0.18	0.836 (0.334−2.092)	0.701
**VIS**	0.057	1.058 (1.001−1.119)	0.048 *
**SVRI** (dyn×sec/cm^5^/m^2^)	−0.003	0.997 (0.995−0.999)	0.028 *

VIS, vasoactive–inotropic score; CI, cardiac index; SVRI, systemic vascular resistance index. * Statistical significance was set at *p* < 0.05.

**Table 3:** Comparison of CI, SVRI, VIS, and ROC at six-hour intervals between survivors and non-survivors.

	CI (L/min/m^2^)			SVRI (dyn×sec/cm^5^/m^2^)			VIS		
Hour	Survival	28-Day Mortality	AUC	Survival	28-Day Mortality	AUC	Survival	28-Day Mortality	AUC
0	3.9 ± 1.2	4.1 ± 1.3	0.51	1413 ± 537	933 ± 376	0.8	28 ± 13	58 ± 55	0.76
6	3.8 ± 1.2	4 ± 1.1	0.46	1543 ± 645	1019 ± 354	0.76	36 ± 22	69 ± 62	0.73
12	3.9 ± 1	4.4 ± 1.2	0.32	1594 ± 552	935 ± 261	0.89	38 ± 26	66 ± 50	0.71
18	4.1 ± 1.4	4.9 ± 1.3	0.34	1731 ± 583	851 ± 394	0.88	36 ± 26	79 ± 57	0.73
24	4.2 ± 1.5	3.9 ± 1	0.47	1697 ± 483	1040 ± 386	0.84	25 ± 14	60 ± 35	0.79
30	4.1 ± 1.1	4.2 ± 0.9	0.44	1602 ± 511	957 ± 322	0.83	23 ± 16	52 ± 32	0.79
36	4.4 ± 1.3	4.1 ± 0.9	0.54	1631 ± 488	1099 ± 551	0.79	22 ± 16	46 ± 27	0.76
42	3.7 ± 1.2	3.6 ± 0.8	0.51	1886 ± 627	1290 ± 658	0.77	21 ± 15	49 ± 28	0.79
48	3.8 ± 1.3	3.7 ± 0.9	0.52	1973 ± 577	1424 ± 613	0.76	21 ± 15	53 ± 36	0.78
54	4 ± 1.3	4.4 ± 1.3	0.38	1768 ± 554	1345 ± 615	0.62	22 ± 13	39 ± 23	0.73
60	3.8 ± 1.5	4.2 ± 0.9	0.35	1866 ± 639	1149 ± 638	0.8	22 ± 12	45 ± 31	0.73
66	3.4 ± 0.8	3.8 ± 1.2	0.4	1951 ± 564	1405 ± 705	0.75	21 ± 13	39 ± 29	0.70
72	3.7 ± 1.1	3.9 ± 1.4	0.52	1766 ± 439	1489 ± 880	0.62	21 ± 13	39 ± 29	0.70

CI, cardiac index; SVRI, systemic vascular resistance index; VIS, vasoactive–inotropic score; AUC, area under the ROC curve; ROC, receiver operating characteristic.

**Table 4:** Best cutoff values of hemodynamic parameters at 6 h intervals within 72 h after the PiCCO setup.

Time (Hours)	Hemodynamic Parameters	Cutoff Value	Sensitivity	Specificity	LR^+^	LR^−^	Youden Index
0	SVRI	896	0.61	0.93	9.2	0.4	0.5
6	SVRI	1011	0.69	0.86	4.9	0.4	0.5
12	SVRI	978	0.77	0.92	10	0.3	0.7
18	SVRI	1294	0.91	0.77	3.9	0.1	0.7
24	SVRI	1284	0.73	0.75	2.9	0.4	0.5
30	SVRI	1014	0.6	0.9	6.6	0.4	0.5
36	SVRI	1446	0.89	0.67	2.7	0.1	0.6
42	VIS	49	0.56	1	-	0.4	0.6
48	VIS	44	0.63	1	-	0.3	0.6
54	VIS	42	0.58	1	-	0.4	0.6
60	SVRI	1312	0.7	0.8	3.5	0.4	0.5
66	SVRI	1454	0.67	0.8	3.3	0.4	0.6
72	VIS	27	0.67	0.78	3	0.4	0.4

LR^+^, positive likelihood ratio; LR^−^, negative likelihood ratio.

**Table 5:** Two cutoff values for SVRI in predicting clinical outcomes with 100% specificity and 100% sensitivity at 6 h intervals.

Time (Hours)	SVRI	Sensitivity	Specificity	LR^+^	LR^−^	Youden Index
0	533	20%	100%	−	0.8	0.2
0	1531	100%	10%	1.1	0	0.1
6	1011	70%	100%	−	0.3	0.7
6	1789	100%	40%	1.7	0	0.4
12	837	60%	100%	−	0.4	0.6
12	1492	100%	70%	3.3	0	0.7
18	914	60%	100%	−	0.4	0.6
18	1534	100%	70%	3.3	0	0.7
24	1156	60%	100%	−	0.4	0.6
24	1590	100%	70%	3.3	0	0.7
30	959	60%	100%	−	0.4	0.6
30	1646	100%	70%	3.3	0	0.7
36	1004	40%	100%	−	0.6	0.4
36	2390	100%	10%	1.1	0	0.1
42	1033	50%	100%	−	0.5	0.5
42	2400	100%	30%	1.4	0	0.3
48	1100	40%	100%	−	0.6	0.4
48	2487	100%	40%	1.7	0	0.4
54	997	30%	100%	−	0.7	0.3
54	2000	100%	20%	1.3	0	0.2
60	1063	60%	100%	−	0.4	0.6
60	2327	100%	40%	1.7	0	0.4
66	1106	50%	100%	−	0.5	0.5
66	2494	100%	40%	1.7	0	0.4
72	1151	50%	100%	−	0.5	0.5
72	2795	100%	0	1	0	0

## Data Availability

The datasets used and analyzed during the current study are available from the corresponding author on reasonable request.
